# Detecting vulnerable carotid plaque and its component characteristics: Progress in related imaging techniques

**DOI:** 10.3389/fneur.2022.982147

**Published:** 2022-09-14

**Authors:** Shi-Ting Weng, Qi-Lun Lai, Meng-Ting Cai, Jun-Jun Wang, Li-Ying Zhuang, Lin Cheng, Ye-Jia Mo, Lu Liu, Yin-Xi Zhang, Song Qiao

**Affiliations:** ^1^The Second Clinical Medical College, Zhejiang Chinese Medicine University, Hangzhou, China; ^2^Department of Neurology, Zhejiang Hospital, Hangzhou, China; ^3^Department of Neurology, Second Affiliated Hospital, School of Medicine, Zhejiang University, Hangzhou, China

**Keywords:** carotid artery atherosclerosis, vulnerable plaque, plaque component characteristics, invasive imaging, non-invasive imaging

## Abstract

Carotid atherosclerotic plaque rupture and thrombosis are independent risk factors for acute ischemic cerebrovascular disease. Timely identification of vulnerable plaque can help prevent stroke and provide evidence for clinical treatment. Advanced invasive and non-invasive imaging modalities such as computed tomography, magnetic resonance imaging, intravascular ultrasound, optical coherence tomography, and near-infrared spectroscopy can be employed to image and classify carotid atherosclerotic plaques to provide clinically relevant predictors used for patient risk stratification. This study compares existing clinical imaging methods, and the advantages and limitations of different imaging techniques for identifying vulnerable carotid plaque are reviewed to effectively prevent and treat cerebrovascular diseases.

## Introduction

Stroke has now become the second leading cause of death worldwide and the first leading cause of death in China (1). Associated with high disability and mortality rates, stroke is an important public health issue that can significantly and negatively impact society (1). Atherosclerosis accounts for approximately 25% of all ischemic strokes, being the plaques of the internal carotid artery the most frequently involved in stroke pathogenesis (2, 3). It is well known that the degree of stenosis is the most important and validated vulnerability biomarker to predict the risk of ipsilateral cerebrovascular events. But with the gradual in-depth research, we realized that the degree of stenosis alone is not sufficient to decide upon which is the best preventive treatment in some important subgroups of patients with carotid atherosclerosis, and that it may be helpful to focus on the characterization of atherosclerotic plaques (2). As a result, this review will focus on the techniques employed to image carotid plaque, describing how some methods are suitable for evaluating specific carotid plaque components.

## Histopathological characteristics of vulnerable plaques

Atherosclerosis (AS) is a complex and progressive inflammatory disease, whose development involves accumulation of lipids in the arteries, formation of foam cells, local inflammatory responses, and migration and proliferation of macrophages, smooth muscle cells, lymphocytes, and neutrophils (3). AS mainly occurs in the intima of medium and large arteries and is mostly located at turbulence and shear stress reduction locations, such as the bend and bifurcation of vessels (4). Vulnerable carotid plaque refers to AS plaque with rupture tendency and easy-to-cause thrombosis, making the patient susceptible to acute cerebrovascular events. This phenomenon is now well described as thin-cap fibroatheroma (TCFA), classified by plaques consisting of lipid-rich necrotic cores (LRNC) and thin fibrous caps (FCs) (<65 μm) infiltrating macrophages and inflammatory cells. As a precursor lesion of plaque rupture, TCFA may share several plaque features associated with increased risk of cerebrovascular events, including inflammation, microcalcification, spot calcification, bleeding, neovascularization, and extraverted remodeling (5, 6).

Naghavi M et al. proposed the histopathological diagnostic criteria for vulnerable plaques in (7, 8), which has now reached a consensus (Table 1). The main diagnostic criteria include inflammatory activation in plaque (monocyte/macrophage or with T lymphocyte infiltration), thin FC (thickness <65 μm) and large lipid core (>40% of the plaque area), vascular endothelial cell ablation with platelet aggregation on the surface, fissures or damaged plaques, and severe luminal stenosis (>90%). Secondary diagnostic criteria include intraplaque hemorrhage (IPH), endothelial dysfunction, superficial nodules or calcifications, yellow plaques, and positive remodeling of vascular walls.

**Table 1:** Histopathological diagnostic criteria for vulnerable plaques.

	**Pathological features**
Main criteria	Inflammatory activation in plaque (monocyte/macrophage or with T lymphocyte infiltration)
	Thin FC (thickness <65 μm)
	Large lipid core (>40% of the plaque area)
	Vascular endothelial cell ablation with platelet aggregation on the surface
	Fissures or damaged plaques
	Severe luminal stenosis (>90%)
Secondary criteria	Intraplaque hemorrhage (IPH)
	Endothelial dysfunction
	Superficial nodules or calcifications
	Yellow plaques
	Positive remodeling of vascular walls

## Imaging identification of vulnerable carotid plaque

Determining plaque characteristics is important for understanding the pathophysiological process of atherosclerotic plaque formation, hence providing us with possible methods for assessing the risk of patient-specific, individual plaques. Therefore, imaging modalities are required to reliably assess plaque composition, thus allowing the implantation of therapeutic strategies to prevent adverse vascular events.

### Non-invasive imaging

#### Ultrasound

B-mode ultrasonography and contrast-enhanced ultrasonography (CEUS) are the two most widely used techniques for assessing carotid plaques (9). B-mode ultrasound images are used to evaluate plaque echoes, which are the ultrasonic equivalent of LRNC. Up to 50% of symptomatic plaques have echoes, compared to less than 5% of asymptomatic plaques (10). Additionally, regardless of stenosis, patients with echogenic plaques were associated with a stroke risk of up to 13%, higher than the risk of stroke in patients with high stenosis (11). Although B-mode carotid ultrasound has high specificity, it has only moderate sensitivity in identifying plaque surface ulcers, and the incidence of detecting symptomatic plaque ulcers varies greatly (12). Moreover, the sensitivity of patients with moderate stenosis is particularly poor (13). Another limitations of B-ultrasound in detecting carotid plaque is the lack of consistency between operators and poor signal-to-noise ratio.

Recent advances in the characterization of carotid plaques *via* ultrasound have also emerged in CEUS field. CEUS uses intravenous microbubble contrast agents. This contrast agent does not diffuse into surrounding tissues as other contrast agents do, so all signals from CEUS examinations are intravascular, allowing accurate assessment of lumen and neovascularity within carotid plaques (14). For carotid stenosis, CEUS can distinguish between complete carotid occlusion and high stenosis, identify plaque ulcers, and assess carotid plaque neovascularization (14). Camps-Renom P et al. found that in patients with an anterior circulation ischaemic stroke and carotid atherosclerosis, plaque neovascularization detected with CEUS was an independent predictor of stroke recurrence (15). This implies that neovascularization is associated with the risk of recurrent stroke, and CEUS can be used as an intermediate measure. However, there are limitations to CEUS use. This technique is highly operator dependent and prone to high inter-observer variability (16). Furthermore, CEUS is prone to enhanced artifacts. The ultrasonic wave propagates through the contrast agent, resulting in an increase in the signal of the vessel wall farthest from the probe, leading to an over-interpretation of the vessel wall enhancement (14).

#### Computed tomography angiography

Computed tomography angiography (CTA) is a non-invasive imaging alternative that can assess vessel wall size, high-risk plaque load, morphological characteristics, and vulnerability with relative accuracy (17). CTA allows high-risk plaques to be classified as calcified, non-calcified, or partially calcified. However, numerous studies have revealed that CTA has limited accuracy in distinguishing lipids, fibrotic tissue components, and intraplaque inflammation due to limited imaging resolution (18). Plaque calcification, fibrous plaque thickness, IPH, and LRNC can be characterized on multidetector-row CT (MDCT) based on voxel Hounsfield units. This technique has also been highly effective in detecting plaque ulcers and plaque neovascularization, with sensitivity and specificity exceeding 90% (19). Unfortunately, there is considerable overlap between the CT densities of LRNC, fibrous tissue, and IPH, resulting in reduced reliability in pixel by pixel assessment of plaque composition (9).

#### High-resolution magnetic resonance imaging

High-resolution magnetic resonance imaging (HRMRI) is currently considered the most competitive imaging method for examining the carotid artery wall due to an extremely high soft-tissue resolution (20). With the application of 3.0T high-field magnetic resonance instruments and the maturation of black-blood technology and multi-contrast sequence imaging technology, MRI offers further advantages to other imaging methods, providing greater aid for identifying plaque internal components and evaluation of plaque stability. Black-blood technology, the core technology of high-resolution MR examination of carotid plaque, can cover the duration of blood flow signal in the carotid artery lumen and the adipose tissue signal outside the carotid wall (21). This allows for clear visualization of the size and shape of carotid plaque, making it easier to distinguish different components in the plaque according to differences in signal intensity, such as LRNC, calcification, and IPH. Various plaque analysis software can also be used to further evaluate the status of the FC, quantitatively analyze plaque composition, and determine whether there is an active inflammatory response in the plaque (22, 23). Serfaty et al. have proposed and published an American Heart Association (AHA) classification of atherosclerotic plaques specifically for MRI. This classification (Table 2) highlights the ability of MR imaging to detect plaques based on composition and morphology. In addition, magnetization-prepared rapid acquisition with gradient-echo (MPRAGE) can display plaque bleeding with high specificity (24, 25). Over the past 5 years, a series of studies has demonstrated that 3.0T high-resolution MR carotid plaque imaging is highly consistent with histopathological results (24). MRI is excellent at detecting and distinguishing between LRNC and IPH. It is also sensitive in detecting plaque ulceration and calcification. Meta-analyses showed a significant positive relationship between IPH and the risk of future ischemic events (HR: 5.69, 95% CI: 2.98–10.87) (26, 27). Over a median follow-up of 19.6 months, the presence of IPH was associated with a six-fold higher risk for cerebrovascular ischemic events (27). HRs for LRNC and TRFC as predictors of subsequent stroke/transient ischemic attack were (3.00, 95% CI: 1.51–5.95) and (5.93, 95% CI: 2.65–13.20), respectively (28). These reports demonstrate that MRI can identify patients at higher (or lower) risk of stroke. However, a downside to MRI is that it can take longer than other modalities, and patients with claustrophobia and metallic devices (such as pacemakers, defibrillators, and certain aneurysm clips) must be excluded from MRI procedure.

**Table 2:** AHA classification criteria for atherosclerotic plaque for MRI.

**Lesion type**	**Characters**
Type I-II	Close to normal wall thickness and no calcification
Type III	Diffuse intimal thickening or small eccentric plaques without calcification
Type IV-V	There is a lipid/necrotic core surrounded by fibrous tissue that may be associated with calcification
Type VI	Complex plaques may be associated with surface damage, bleeding, or thrombosis
Type VII	Calcified plaque
Type VIII	Fibrous plaques without a necrotic core may be accompanied by microcalcification

#### Positron emission tomography

Positron emission tomography (PET) is a non-invasive nuclear imaging technique that uses tracers to assess the biological processes associated with AS, such as intraplaque inflammation, microcalcification, and intraplaque angiogenesis. ^18^F-fluorodeoxyglucose (^18^F-FDG) is a radioactively labeled glucose analog that can be absorbed by metabolically active cells, such as macrophages, and is the most commonly used tracer in PET imaging (29). Several studies on human and animal models of AS have shown that ^18^F-FDG can selectively aggregate in activated macrophages demonstrating plaque active inflammation, as macrophage density is a histological indicator of vascular inflammation (29–31). In addition, FDG-PET indicates common cardiovascular risk factors, with significant correlation observed for factors such as obesity, gender (male), age (>65 years), smoking, hypertension, diabetes, and hypercholesterolemia. Local arterial inflammation exists in patients with these risk factors, indicating the significant predictive value that FDG-PET imaging can have in disease progression (32). In a multicenter prospective cohort study of patients with carotid stenosis and recent stroke/transient ischemic attack, Kelly et al. showed that plaque inflammation associated ^18^F-FDG uptake independently predicted future recurrent stroke after PET (33). Similarly, in a PET study using ^18^F-fluorcholine (^18^F-FCH), histological analysis of carotid endarterectomy specimens revealed a strong correlation between the uptake of the tracer and the macrophage infiltration (34, 35). Other examples of PET include ^68^Ga-DOTATATE PET (36), used in the expression of somatostatin receptor imaging, as the tool of detecting artery inflammation, or CD80-targeting PET Tracers (37). These techniques offer non-invasive detection of inflammation at high spatial resolution. However, PET imaging has its limitations. With a spatial resolution of approximately 6 mm, the direct quantification of vulnerable plaques in smaller vessels is limited. Moreover, the cost and availability of PET are limited due to radiation exposure (3).

Developing novel PET tracers is an active area of in-depth research. These novel tracers include ^18^F-FMISO, ^68^GA-NOTA-RGD, and ^18^F-NAF (38, 39). ^18^F-NAF, in particular, is a tracer for active microcalcification in atherosclerotic plaques, which could be useful in identifying vulnerable carotid plaques. Clinical trials conducted by Bekaert L et al. and Thureau S et al. demonstrated that ^18^F-FMISo is associated with angiogenesis and can be used as a marker of angiogenesis (40, 41). Furthermore, Kim et al. demonstrated that ^68^GA-NOTA-RGD is a reliable tracer in angiogenic imaging (42). Nevertheless, these radioactive tracers must be validated in more large clinical studies.

### Invasive imaging

#### Intravascular ultrasound

Intravascular ultrasound (IVUS) is a catheter-based imaging method delivering acoustic waves into the patient *via* a transducer or probe. In gray-scale IVUS, the signal from these acoustic waves is backscattered and processed into a two-dimensional video image in real-time, displaying different components such as fibers, calcification, and lipid nucleus. With IVUS, it is also possible to determine the size and distribution of vascular walls and the severity of atherosclerotic plaque present (3). An even more innovative approach to IVUS is virtual histology-intravascular ultrasound (VH-IVUS), an analysis technology allowing for real-time cross-sectional and longitudinal three-dimensional visualization of the vessel. The technology benefits from advanced radiofrequency analysis of reflected echo signals to generate multiple spectral parameters, using mathematical operations to convert the signals into color histograms for analyzing different plaque components (43). Using these techniques, VH-IVUS can then classify plaque into the four groups of fibrous plaque, fibrolipid plaque, necrotic core, and dense calcium, providing a morphological evaluation of lesion evolution (44, 45). In a prospective multicenter registry (VICTORY) study, IVUS and IVUS-VH examinations during carotid artery interventional therapy were determined both feasible and safe, providing important insights into the qualitative and quantitative composition of carotid plaques (46). In a study conducted by Diethrich et al., it was found that a strong correlation between VH IVUS plaque characterization and the true histological examination of the plaque following endarterectomy, particularly in “vulnerable” plaque types. And the diagnostic accuracy of VH IVUS to agree with true histology in different carotid plaque types was 99.4% in TCFA, 96.1% for calcified TCFA, 85.9% in fibroatheroma, 85.5% for fibrocalcific, 83.4% in pathological intimal thickening, and 72.4% for calcified fibroatheroma (47).

Despite the increased use of VH-IVUS in clinical studies to identify vulnerable plaque features, its ability to identify and quantify TCFA has been challenged. PROSPECT (NCT00180466) (Providing Regional Observations to Study Predictors of Coronary Tree) study reported that 697 patients with acute coronary syndrome underwent conventional angiography, grayscale IVUS, and VH-IVUS, IVUS was unable to visualize the entire coronary tree. It was also reported that only 53% of the lesions leading to adverse cardiovascular events were assessed during a median follow-up of 3.4 years (48). In addition, the multifactorial analysis revealed only 18.2% positive predictive values for detecting susceptibility to major acute coronary events (MACE) during follow-up, with positive defined variables including plaque load >70%, minimum cavity area <4 mm^2^, or, according to VH-IVUS, classified as TCFA (lesions with necrotic core ≥10%, no obvious fibrous tissue covered, and atherosclerotic plaque volume percentage ≥40%) (48).

IVUS-based mode deficiencies may depend on technical constraints, such as operator-related parameters and spatial resolution. VH-IVUS has limited axial resolution (100–200 μm), which hinders the identification of FC thickness (TCFA thickness is less than the spatial resolution of the system), plaque destruction, macrophage infiltration, and thrombosis in plaques (3, 49). Furthermore, microcalcification is a good indicator of rupture susceptibility; however, IVUS only has high sensitivity and specificity for identifying large dense calcified plaques or spot calcifications (49). In summary, IVUS is complex, innovative, and expensive and currently suffers from a low penetration rate.

#### Optical coherence tomography

Optical coherence tomography (OCT) can be considered the optical analog of IVUS. Instead of the acoustic waves used in the ultrasonic examination, OCT works *via* the optical echo of near-infrared light, which has a spatial resolution approximately ten times higher than that of IVUS (10–15μm) (50). Due to the strong light attenuation of blood, the blood vessels must be cleared by injection of a contrast agent to obtain an accurate image. Due to its high spatial resolution, OCT allows quantification of FC thickness (51). However, limited tissue penetration depth (1–2.5 mm) precludes visualization of large plaques or use in large vessels (49). Other vulnerable plaque features seen on OCT include neovascularization, IPH, calcification, and cholesterol crystallization (52). Inflammation of vulnerable plaques can be observed and quantified by measuring macrophage infiltration in the FC (53). Feng et al. found that the ruptured plaque contained more macrophages than the unruptured plaque (6.95 vs. 5.29%, *p* = 0.002) (54). It has also been reported that OCT has high sensitivity and specificity in detecting lipid-rich plaques, verified by autopsy specimens (90–94% and 90–92%, respectively) (55). Another study demonstrated a significant correlation between FC thickness, lipid core size, and the proportion of lipid content between groups through OCT measurement (56), confirming the high efficiency of OCT. Moreover, OCT cap thickness measurements were associated with the prevalence of plaque rupture (57). Unlike IVUS, OCT can penetrate plaque calcification and describe it in detail in terms of thickness, area, and volume (3). It is well known that extensive calcification is associated with stable plaques. Conversely, in contrast to large calcifications, the presence of microcalcifications detected by IVUS or OCT corresponds to plaque instability. Recent OCT findings suggest that the co-location of macrophages and microcalcifications within the same plaque is associated with a higher degree of plaque vulnerability. In such cases, the same patients showed less advanced arterial stenosis, indicating that the co-location of macrophages and microcalcifications suggests an early stage of the atherosclerotic process, which may progress to further calcification and inflammation (58, 59). These observations suggest that OCT can ensure the assessment of morphological features and atherosclerotic disease activity. To visualize the microstructure, micro-OCT (μOCT), which can achieve an axial resolution of ≤ 1 μm, was developed (60). Using μOCT enables a deeper understanding of the underlying biological processes of AS by visualizing cellular components and can thus refine and expand the definition of vulnerable plaques.

### Near-infrared spectroscopy

Near-infrared spectroscopy (NIRS) is another intravascular imaging mode, which absorbs and scatters NIR light (wavelengths from 800 to 2,500 nm) at different intensities, depending on the properties of substances as a function of the wavelength (61). NIRS technology was first applied in animal experiments by Cassis and Lodder in 1993, who proved that this model could visualize lipid deposition in the aorta of hypercholesterolemia rabbits (62). In 2002, Moreno et al. first reported NIRS application for detecting LRNC in human aortic specimens (63). Histologically, the authors found that the sensitivity and specificity of lipid pools were 90 and 93%, respectively, and 77 and 93% for thin caps.

Although NIRS can provide a reliable and quantitative assessment of lipinuclear plaques, several significant limitations have prevented NIRS from dominating as an independent method in the wider clinical setting. First, NIRS can only provide lipid composition characteristics; however, it does not support a complete morphological assessment of plaques. Second, visualization and assessment of lumen, external vessel wall size, and plaque load are not achievable with NIRS. Third, it lacks image depth resolution to locate the necrotic core within the plaque and distinguish TCFA from thick hat fibroma.

### Multimodal imaging

To overcome the limitations of the above techniques and enhance their reliability, an intravascular hybrid imaging method combining two different modes has been proposed to assess plaque morphology and comprehensively predict disease progression. Studies have revealed that IVUS-NIRS combined imaging is particularly beneficial for simultaneously identifying the distribution of lipid core plaques and studying the relationship between vascular geometry, shear stress, and plaque composition (64). For example, IVUS alone can detect fibrous atherosclerotic plaques, whose components are often obscured by the presence of calcification. NIRS can detect lipids, regardless of whether they contain several calcifications (65). Several other IVUS-NIRS studies have also demonstrated improvements in IVUS-NIRS fusion efficacy (65). In PACMAN-AMI randomized clinical trial (NCT03067844), the combination of IVUS and NIRS was successfully used to evaluate the effect of statins on plaque burden and composition (66). The ATHEROREMO-IVUS study (NCT01789411) and other recent prospective studies suggest that IVUS-NIRS can be used as a diagnostic tool in clinical practice to detect vulnerable plaques (especially fatty plaques) and patients at high risk for subsequent MACE (67–69). IVUS-NIRS is the only hybrid endovascular imaging technique approved by the US Food and Drug Administration for clinical use due to its great efficacy and availability. However, in addition to the loss of IVUS signals behind calcified tissues described above, limitations of IVUS-NIRS include the low resolution of IVUS, limiting the evaluation of cap thickness, and luminal boundary definitions in the presence of thrombosis or severe intraplaque bleeding.

Alternatively, it has also been recommended to integrate the two approaches of IVUS and OCT, combining the deep penetration of IVUS with the high resolution of OCT. Therefore, a dual-mode IVUS-OCT catheter capable of obtaining OCT and IVUS images was introduced, allowing more precise real-time measurements of FC thickness, necrotic core, and plaque load to be obtained in the *in vitro* specimens and animal models (46, 70). IVUS-OCT application has reportedly demonstrated improved imaging characteristics, providing supplementary information for TCFA detection (71). A prospective cohort study (NCT00617084 NCT00962416) in humans revealed that IVUS-OCT combination is feasible in most patients, with no differences observed in the incidence of MACE between percutaneous coronary intervention patients with and without endovascular imaging during the 2-year follow-up period, confirming the long-term safety of the approach (72).

OCT-NIRS catheters have also been developed to provide OCT and NIRS data in a pull-back, combining NIRS advantage of identifying lipid core components with OCT advantage of determining FC thickness on lipid pools (73). Additionally, further innovative multimodal imaging techniques, such as OCT-near-infrared fluorescence (NIRF) (74), IVUS-NIRF (75), IVUS-intravascular photoacoustic imaging (IVPA) (76), and IVUS-fluorescence lifetime imaging microscopy (FLIM) (77) are currently undergoing preclinical evaluation.

## Future speculation and overall conclusions

Rapid advances in carotid imaging have provided important pathophysiological insights. A growing body of evidence supports using carotid artery imaging to characterize carotid plaque characteristics, including carotid plaque load, plaque composition, endoluminal surface condition, and plaque inflammation and neovascularization. Although none of the above imaging modalities can provide a complete and comprehensive assessment of all plaque vulnerability signs and the mechanisms underlying atherosclerotic progression, advances in invasive and non-invasive imaging techniques have demonstrated noteworthy diagnostic and prognostic value. Extensive imaging and pathology studies are required to observe plaque composition and demonstrate how current technological advances can be transformed from attractive images into imaging strategies that can be widely adopted in clinical settings.

As previously observed, multimodal imaging can optimize the limitations of a single imaging mode in invasive imaging, more comprehensively assess plaque morphology, and predict disease progression. Novel imaging modalities such as OCT-NIRS, OCT-NIRF, IVUS-NIRF, IVPA, and FLIM are currently undergoing preclinical evaluation. We, therefore, look forward to further advances in these studies to more accurately assess carotid vulnerable plaques. In non-invasive imaging, it has been proposed that molecular imaging using novel, targeted nanoparticles may assist in detecting high-risk plaques and even provide non-invasive treatment strategies (78). However, none of these imaging methods can detect vulnerable plaques, nor have they been shown to predict outcomes definitively. Further trials are needed to provide more information regarding high-risk plaques and refine future plaque stabilization strategies. As deep learning technology develops, artificial intelligence (AI) has also been proposed to automatically identify, classify, and quantify the composition of carotid plaques (79). Many studies have applied AI to image acquisition or optimization using post-processing tools, particularly in MRI field (80). Applying AI methods in carotid plaque imaging remains in its infancy, and many innovations in this field are expected in the coming years (81).

## Author contributions

S-TW contributed to the study design of this review. S-TW, Q-LL, M-TC, Y-XZ, and SQ equally performed the literature search and wrote the manuscript. J-JW, L-YZ, LC, Y-JM, and LL profoundly enriched the manuscript by adding important intellectual content. All authors contributed to the article and approved the submitted version.

## Funding

This work was supported by the Scientific Research Fund of the National Health and Family Planning Commission [No. WKJ-ZJ-1809] and Program of Zhejiang Provincial Science and Technology Foundation, China [No. 2018C37132].

## Conflict of interest

The authors declare that the research was conducted in the absence of any commercial or financial relationships that could be construed as a potential conflict of interest.

## Publisher's note

All claims expressed in this article are solely those of the authors and do not necessarily represent those of their affiliated organizations, or those of the publisher, the editors and the reviewers. Any product that may be evaluated in this article, or claim that may be made by its manufacturer, is not guaranteed or endorsed by the publisher.
